# Single transcript unit CRISPR 2.0 systems for robust Cas9 and Cas12a mediated plant genome editing

**DOI:** 10.1111/pbi.13068

**Published:** 2019-01-17

**Authors:** Xu Tang, Qiurong Ren, Lijia Yang, Yu Bao, Zhaohui Zhong, Yao He, Shishi Liu, Caiyan Qi, Binglin Liu, Yan Wang, Simon Sretenovic, Yingxiao Zhang, Xuelian Zheng, Tao Zhang, Yiping Qi, Yong Zhang

**Affiliations:** ^1^ Department of Biotechnology Center for Informational Biology School of Life Sciences and Technology University of Electronic Science and Technology of China Chengdu China; ^2^ Jiangsu Key Laboratory of Crop Genetics and Physiology Jiangsu Co‐Innovation Center for Modern Production Technology of Grain Crops Jiangsu Key Laboratory of Crop Genomics and Molecular Breeding College of Agriculture Yangzhou University Yangzhou China; ^3^ Key Laboratory of Plant Functional Genomics of the Ministry of Education Joint International Research Laboratory of Agriculture and Agri‐Product Safety of the Ministry of Education Yangzhou University Yangzhou China; ^4^ Department of Plant Science and Landscape Architecture University of Maryland College Park MD USA; ^5^ Institute for Bioscience and Biotechnology Research University of Maryland Rockville MD USA

**Keywords:** rice, single transcript unit, CRISPR‐Cas9, CRISPR‐Cas12a, base editing

## Abstract

CRISPR‐Cas9 and Cas12a are two powerful genome editing systems. Expression of CRISPR in plants is typically achieved with a mixed dual promoter system, in which Cas protein is expressed by a Pol II promoter and a guide RNA is expressed by a species‐specific Pol III promoter such as U6 or U3. To achieve coordinated expression and compact vector packaging, it is desirable to express both CRISPR components under a single Pol II promoter. Previously, we demonstrated a first‐generation single transcript unit (STU)‐Cas9 system, STU‐Cas9‐RZ, which is based on hammerhead ribozyme for processing single guide RNAs (sgRNAs). In this study, we developed two new STU‐Cas9 systems and one STU‐Cas12a system for applications in plants, collectively called the STU CRISPR 2.0 systems. We demonstrated these systems for genome editing in rice with both transient expression and stable transgenesis. The two STU‐Cas9 2.0 systems process the sgRNAs with Csy4 ribonuclease and endogenous tRNA processing system respectively. Both STU‐Cas9‐Csy4 and STU‐Cas9‐tRNA systems showed more robust genome editing efficiencies than our first‐generation STU‐Cas9‐RZ system and the conventional mixed dual promoter system. We further applied the STU‐Cas9‐tRNA system to compare two C to T base editing systems based on rAPOBEC1 and PmCDA1 cytidine deaminases. The results suggest STU‐based PmCDA1 base editor system is highly efficient in rice. The STU‐Cas12a system, based on Cas12a’ self‐processing of a CRISPR RNA (crRNA) array, was also developed and demonstrated for expression of a single crRNA and four crRNAs. Altogether, our STU CRISPR 2.0 systems further expanded the CRISPR toolbox for plant genome editing and other applications.

## Introduction

Clustered Regularly Interspaced Short Palindromic Repeats (CRISPR)‐Cas (CRISPR associated) is a leading genome editing toolbox. Cas9 is an RNA guided sequence‐specific nuclease (SSN) that mediates DNA targeting. The most popular *Streptococcus pyogenes* Cas9 (SpCas9) recognizes a target site containing an NGG protospacer adjacent motif (PAM) (Jinek *et al*., 2012). Since its demonstration in plants in late 2013 (Li *et al*., 2013; Nekrasov *et al*., 2013; Shan *et al*., 2013), CRISPR‐Cas9 has been widely applied in many plant species (Malzahn *et al*., 2017; Yin *et al*., 2017). Most studies have relied on non‐homologous end joining (NHEJ) DNA repair pathway to introduce insertion or deletion (InDel) mutations, achieving editing outcomes such as gene knockout (Malzahn *et al*., 2017), mutagenesis of microRNAs (Zhou *et al*., 2017) or *cis*‐regulatory sequences (Rodriguez‐Leal *et al*., 2017; Zhang *et al*., 2018), as well as large chromosomal deletions (Zhou *et al*., 2014) and gene replacement (Li *et al*., 2016). In some cases, homology directed repair (HDR) was used for targeted gene replacement (Endo *et al*., 2016b; Gil‐Humanes *et al*., 2017; Li *et al*., 2015; Miki *et al*., 2018; Schiml *et al*., 2014; Svitashev *et al*., 2015). Cas9 nickase (Fauser *et al*., 2014; Ran *et al*., 2013) and base editors (Gaudelli *et al*., 2017; Komor *et al*., 2016; Li *et al*., 2017; Shimatani *et al*., 2017) have further expanded the applications of Cas9‐based plant genome editing (Hua *et al*., 2018; Lowder *et al*., 2018; Lu and Zhu, 2017; Ren *et al*., 2017, 2018; Yan *et al*., 2018; Zong *et al*., 2017).

CRISPR‐Cas12a (formerly Cpf1), a class 2 type V‐A CRISPR‐Cas system, has also been applied for plant genome editing (Begemann *et al*., 2017; Endo *et al*., 2016a; Hu *et al*., 2017; Kim *et al*., 2017; Li *et al*., 2018a,b; Tang *et al*., 2017; Xu *et al*., 2016; Zhong *et al*., 2018). Unlike Cas9, Cas12a only requires CRISPR RNA (crRNA) without the need of trans‐activating crRNA (tracrRNA) and it recognizes T‐rich PAMs, resulting in staggered DNA double strand breaks (DSBs; Fagerlund *et al*., 2015; Zetsche *et al*., 2015). In addition, Cas12a has ribonuclease activity that helps process the crRNA to maturity (Fonfara *et al*., 2016; Zetsche *et al*., 2017), which has been conveniently utilized for multiplexed plant genome editing (Wang *et al*., 2017b, 2018a). Despite many characteristic differences, Cas9 and Cas12a were both found to be very specific in mediating plant genome editing either by whole‐genome sequencing (Tang *et al*., 2018) or by CIRCLE‐seq (Lee *et al*., 2018; Tsai *et al*., 2017). In general, Cas9 and Cas12a have been demonstrated as highly efficient and specific SSNs in plants.

Most CRISPR‐Cas9 studies used a mixed dual promoter system in which Cas9 is expressed by a Pol II promoter and the single guide RNA (sgRNA) is expressed by a Pol III promoter such as U6 or U3. While it is relatively easy to deploy CRISPR‐Cas9 for multiplexed genome editing, stacking multiple sgRNA expression units quickly adds up to the length of an expression vector (Lowder *et al*., 2015; Ma *et al*., 2015; Xing *et al*., 2014; Zhang *et al*., 2016). It is challenging to package all components into a virus‐based delivery vector (Ali *et al*., 2015; Baltes *et al*., 2014; Cody *et al*., 2017). In addition, repetitive use of multiple U6 or U3 promoters within one construct may cause variations on sgRNA expression levels and transgene silencing in plants (Ma *et al*., 2015). A lot of efforts have been put into development of a compact sgRNA expression system where multiple sgRNAs can be expressed from a single Pol III or Pol II promoter. For example, multiple sgRNAs can be expressed from a single Pol III promoter or a single Pol II promoter when spaced with tRNAs (Cermak *et al*., 2017; Xie *et al*., 2015). Alternatively, sgRNAs can be processed by hammer head (HH) and hepatitis delta virus ribozymes (He *et al*., 2017) and Csy4 RNA ribonuclease (Cermak *et al*., 2017; Tsai *et al*., 2014). Among these CRISPR‐Cas9 expression systems, Cas9 and sgRNAs are generally expressed in two separate expression units. Since Pol III promoters in many organisms are not well characterized and such promoters are typically more suitable to express short transcripts, it is advantageous to use Pol II promoters to express multiple sgRNAs or crRNAs for multiplexed genome editing. Furthermore, higher genome editing efficiencies with Cas9 and Cas12a have been observed with selected constitutive Pol II promoters when compared to Pol III promoters (e.g. U6 or U3) in plants (Cermak *et al*., 2017; Mikami *et al*., 2017; Tang *et al*., 2016) and mammalian cells (Zhong *et al*., 2017). The use of Pol II promoters will also render guide RNAs under spatiotemporal control, enabling more sophisticated applications such as transcriptional regulation (Lowder *et al*., 2015, 2018; Tang *et al*., 2017).

To achieve most simplified, compact and coordinated expression configuration, it is desirable to express the Cas gene and the guide RNAs from a single Pol II promoter. We previously demonstrated one such single transcript unit (STU) system, STU‐Cas9‐RZ, in which Cas9 and sgRNAs were linked by a poly A sequence and sgRNAs were processed by the HH ribozyme (Tang *et al*., 2016). While the HH ribozyme system seems self‐sufficient for sgRNA processing, it has potential drawbacks of low *in vivo* processing activity (Mikami *et al*., 2017) and may not be suitable for expression in a virus‐derived vector (Cody *et al*., 2017). In this study, we sought to develop improved STU systems that are highly efficient for plant genome editing while overcoming the potential drawbacks of the STU‐Cas9‐RZ system. Using rice as a test platform, two STU‐Cas9 systems based Csy4 and tRNA were developed and closely compared with the STU‐Cas9‐RZ system for targeting one, two or three sites using protoplast transformation and stable transformation systems. The promising STU‐Cas9‐tRNA system was further demonstrated for high capacity multiplexed genome editing as well as targeted C to T base editing. Finally, we developed a STU‐Cas12a system and demonstrated its effectiveness for genome editing in rice. We called these new systems collectively as the STU CRISPR 2.0 systems.

## Results

### Comparison of three STU‐Cas9 systems in rice cells

**Figure 1:**
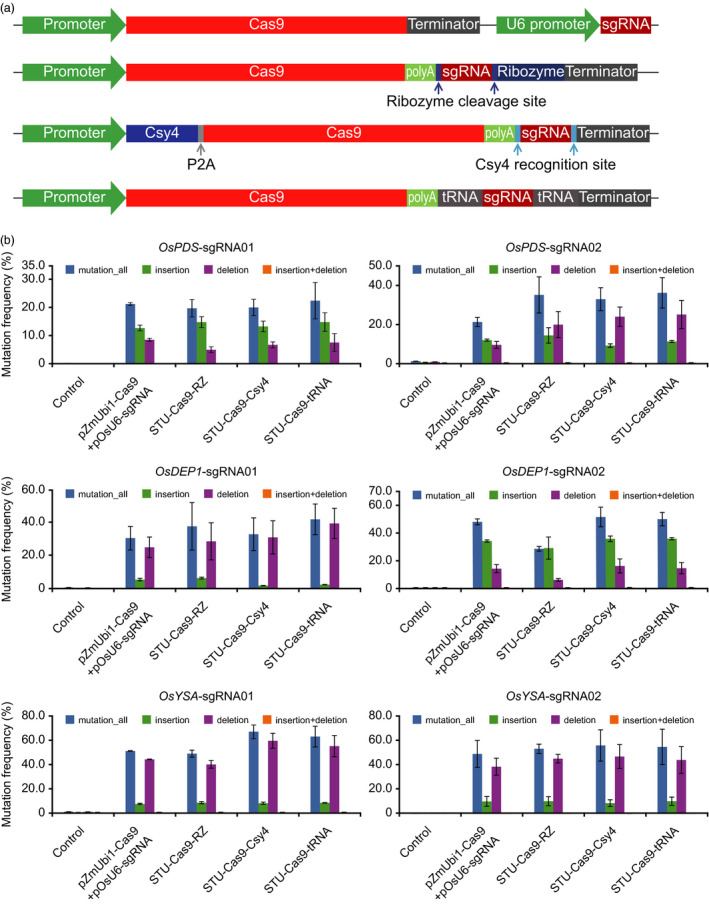
Comparison of three STU‐Cas9 systems in rice cells. (a) Schematics of four CRISPR‐Cas9 expression systems: conventional dual promoter system, STU‐Cas9‐RZ system, STU‐Cas9‐Csy4 system, and STU‐Cas9‐tRNA system. (b) Mutation frequencies at six target sites by four Cas9 systems. At each target site, total NHEJ mutations were also broken down to insertions, deletions as well as insertions plus deletions. The experiments were carried out in rice protoplasts and the frequencies were measured by amplicon‐based deep sequencing. Error bars represent standard deviations of two biological replicates.

Our previous STU‐Cas9‐RZ system utilized HH ribozyme for sgRNA processing (Tang *et al*., 2016). To develop second‐generation STU‐Cas9 2.0 systems, we decided to use the endoribonuclease Csy4 and tRNA for sgRNA processing for two reasons. First, these two systems rely on different mechanisms that are distinct from ribozyme: Csy4 is originated from a bacterial CRISPR transcript (pre‐crRNA) processing system (Haurwitz *et al*., 2010) and the tRNA system relies on the plant endogenous tRNA‐processing system (Xie *et al*., 2015). Second, efficient genome editing has been demonstrated in diverse eukaryotic organisms with Csy4‐based sgRNA processing (Cermak *et al*., 2017; Ferreira *et al*., 2018; Qin *et al*., 2015; Tsai *et al*., 2014) and tRNA‐based sgRNA processing (Port and Bullock, 2016; Shiraki and Kawakami, 2018; Wu *et al*., 2018; Xie *et al*., 2015). Hence, there is a good chance that a STU‐Cas9 2.0 system (STU‐Csy4 or STU‐tRNA) may outperform the STU‐Cas9‐RZ system. The three STU‐Cas9 systems were expressed from the same Pol II promoter, maize ubiquitin promoter (pZmUbi), and compared to the conventional mixed dual promoter system (Figure 1a). For STU‐Cas9‐Csy4, a P2A ribosomal skipping peptide (Szymczak *et al*., 2004) was used to translate Csy4 and Cas9 from a single transcript (Figure 1a). With these four Cas9 systems, we targeted six sites in the rice genome. The resulting 24 constructs were used for transient transformation of rice protoplasts. NHEJ mutations were detected in all these samples by cleaved amplified polymorphic sequence (CAPS) analysis (Figure S1). Mutation frequencies, as sums of insertion and deletions at the target sites, were measured by deep sequencing of polymerase chain reaction (PCR) amplicons. Nucleotide substitutions were rare, and they were excluded for calculation of mutation frequencies since we could not distinguish them from sequencing errors. The three STU systems showed similar editing efficiencies at four target sites: *OsPDS*‐sgRNA01, *OsPDS*‐sgRNA02, *OsYSA*‐sgRNA02 and *OsDEP1*‐sgRNA01 (Figure 1b). However, at *OsYSA*‐sgRNA01 and *OsDEP1*‐sgRNA02 sites, higher editing efficiencies were observed for STU‐Cas9‐Csy4 and STU‐Cas9‐tRNA, when compared to STU‐Cas9‐RZ (Figure 1b). Across all six sites, STU‐Cas9‐Csy4 and STU‐Cas9‐tRNA had the same or higher editing efficiencies when compared to the conventional mixed dual promoter system (Figure 1b). These results suggest that STU‐Cas9‐Csy4 and STU‐Cas9‐tRNA are more robust in genome editing than the first‐generation STU‐Cas9‐RZ system.

**Figure 2:**
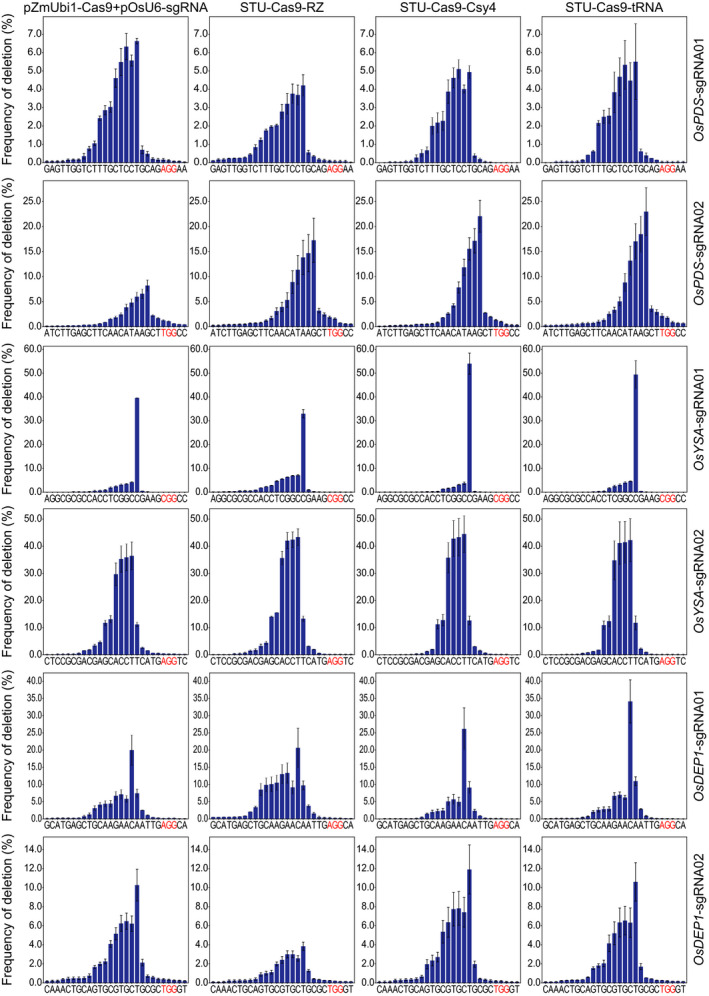
Comparison of positional deletion frequencies at six target sites. The experiments were carried out in rice protoplasts and the frequencies were measured by amplicon‐based deep sequencing. The PAM sites are highlighted in red. Each line represents the same target site, while each column represents the same Cas9 expression strategy. Error bars represent standard deviations of two biological replicates.

**Figure 3:**
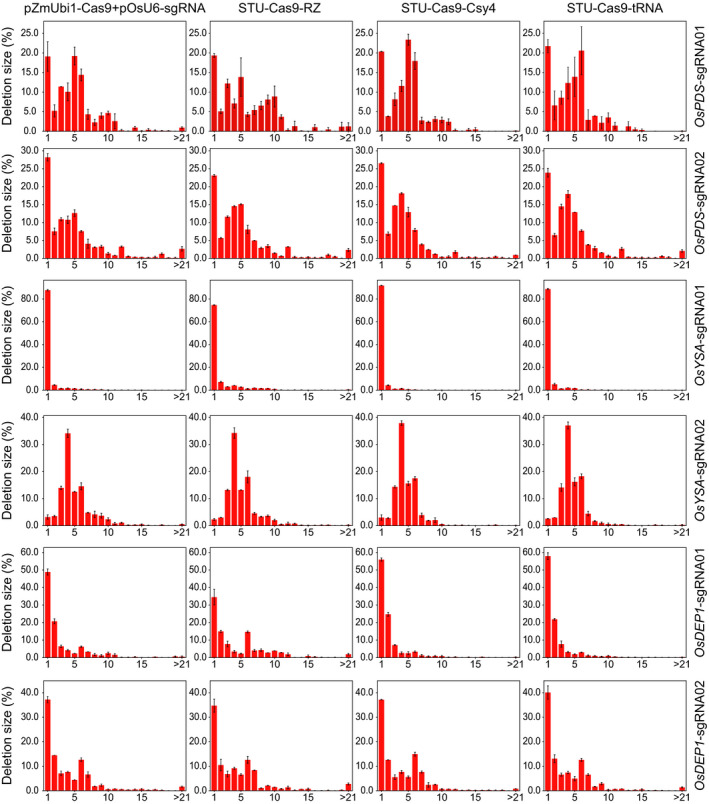
Frequencies of deletions of different sizes at six target sites. The experiments were carried out in rice protoplasts and the frequencies were measured by amplicon‐based deep sequencing. Each line represents the same target site, while each column represents the same Cas9 expression strategy. Error bars represent standard deviations of two biological replicates.

We analysed the NHEJ mutations among all samples to further investigate the editing outcomes of different Cas9 expression systems. The results revealed that the deletion profiles varied greatly across the target sites (Figure 2). However, four different expression systems resulted in very similar deletion profiles, suggesting the NHEJ repair outcomes are largely dictated by the sequence composition of the target sites and but not influenced by the expression systems (Figure 2). Cas9 generates DNA DSBs with mostly blunt ends at 3 bp upstream from the NGG PAM. Interestingly, the most frequent deletion positions were at 4 or 5 bp upstream of the PAM site (Figure 2). Also, deletion profiles at all six sites showed a rather asymmetric distribution where the deletion frequencies for positions between the DNA DSB and the PAM drastically dropped, and there were only few occasions that the PAM sites got deleted (Figure 2). The distribution of deletion size at these target sites further demonstrated that the NHEJ outcomes varied across target sites (Figure 3). While 1 bp deletions were the most predominant deletion type, deletions of more than 3 bp were also very common (Figure 3). These data suggest NHEJ repair outcomes are heavily dependent on target sequence composition, implying microhomology based alternative NHEJ (altNHEJ) is frequently used for repair of Cas9 induced DSBs.

### Comparison of three STU‐Cas9 systems in rice transgenic lines

**Figure 4:**
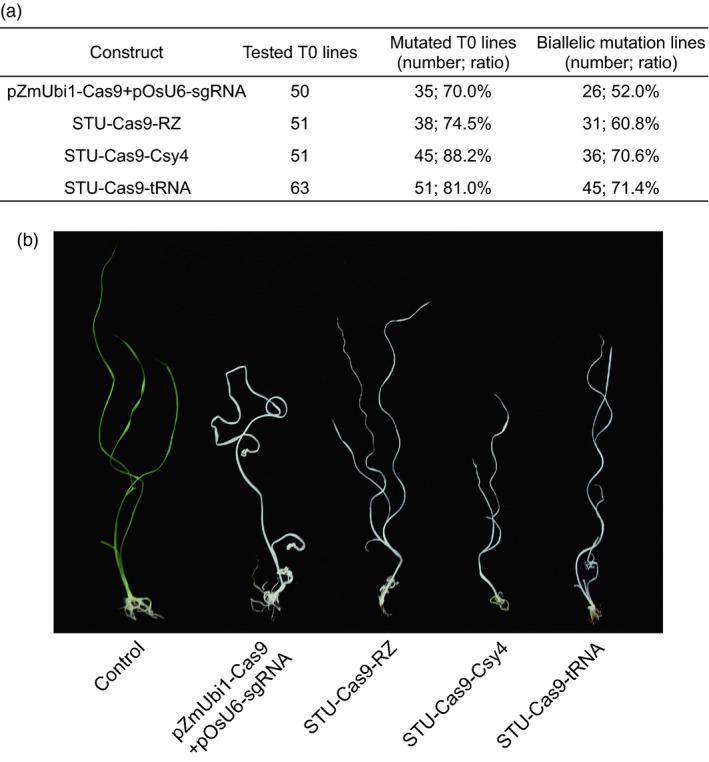
Evaluation of three STU‐Cas9 systems with OsPDS‐sgRNA01 in rice transgenic lines. (a) Mutation rates in stable transgenic T0 lines. Each line was genotyped by Sanger sequencing of PCR amplicons. (b) Phenotype of wild type control and example lines that contain biallelic mutations at the *OsPDS* target site.

We further compared the three STU‐Cas9 systems with the mixed dual promoter system in stable transgenic rice plants. We focused on the *OsPDS*‐sgRNA01 for testing editing efficiency of the four expression systems (Figure 4a). For each construct, 50 or more T0 transgenic plants were obtained for genotyping with Sanger sequencing. With the mixed dual promoter system, 35 out of 50 (70%) T0 plants were mutated and 26 plants (52%) carried biallelic mutations. For STU‐Cas9‐RZ system, 38 out of 51 (74.5%) were mutated and 31 plants (60.8%) carried biallelic mutations. For STU‐Cas9‐Csy4 system, 45 out of 51 (88.2%) were mutated of 36 plants (70.6%) carried biallelic mutations. For STU‐Cas9‐tRNA system, 51 out of 63 (81%) were mutated and 45 plants (71.4%) carried biallelic mutations. Because knockout of *OsPDS* results in albino phenotype, we indeed observed the albino phenotype among all biallelic mutants (Figure 4b). These results indicated the STU‐Cas9‐Csy4 and STU‐Cas9‐tRNA systems are more robust than the STU‐Cas9‐RZ and the mixed dual promoter system for targeted mutagenesis in rice. Hence, the results from stable transgenic lines are consistent with those from rice protoplasts.

### Comparison of three STU‐Cas9 systems for multiplexed editing of two and three sites

**Figure 5:**
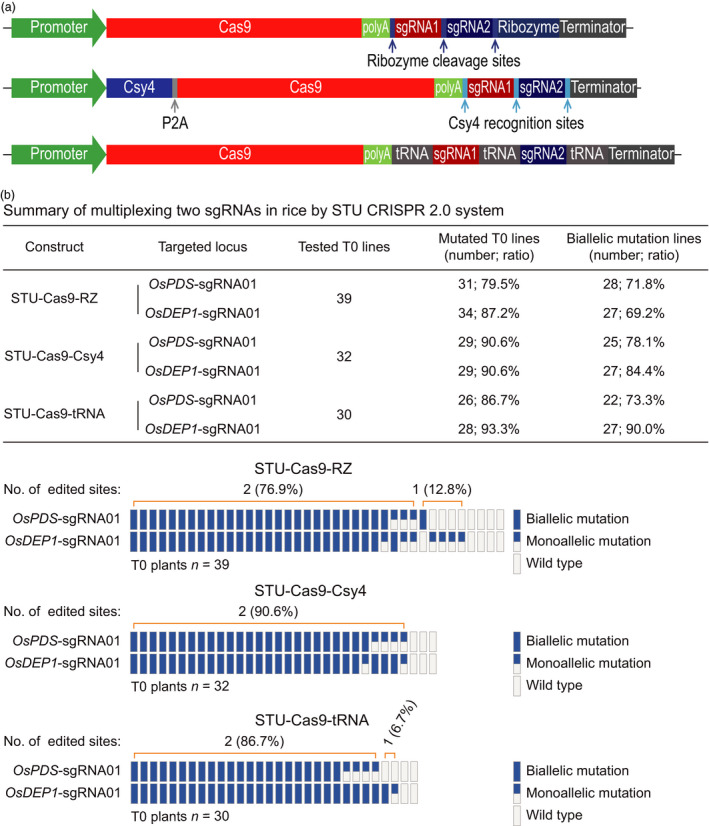
Evaluation of three STU‐Cas9 systems with multiplexed two sgRNAs in rice transgenic lines. (a) Schematics of the STU‐Cas9 expression constructs. (b) Upper panel: a summary table of mutation frequencies at two target sites by three STU‐Cas9 systems; lower panel: a schematic presentation of the genotyping results for all T0 lines categorized as biallelic mutation, monoallelic mutation and wild type.

**Figure 6:**
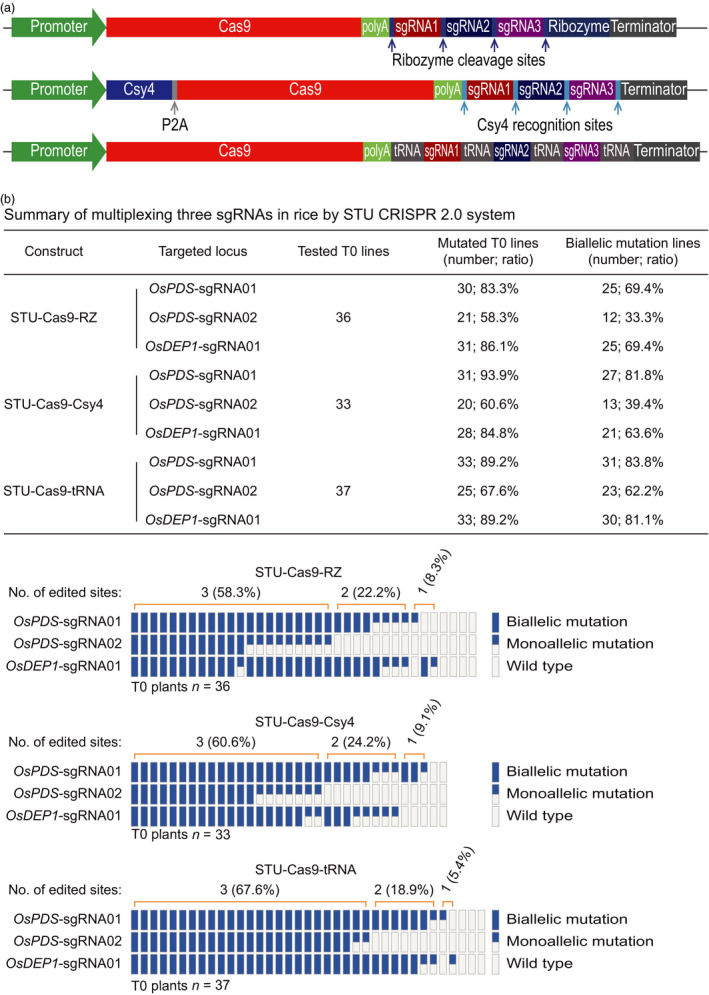
Evaluation of three STU‐Cas9 systems with multiplexed three sgRNAs in rice transgenic lines. (a) Schematics of the STU‐Cas9 expression constructs. (b) Upper panel: a summary table of mutation frequencies at three target sites by three STU‐Cas9 systems; lower panel: a schematic presentation of the genotyping results for all T0 lines categorized as biallelic mutation, monoallelic mutation, and wild type.

We next compared the three STU‐Cas9 systems for multiplexed editing using the rice stable transformation system. First, we simultaneously targeted two sites: *OsPDS*‐sgRNA01 and *OsDEP1*‐sgRNA01 (Figure 5a). For each construct, over 30 independent T0 lines were genotyped by Sanger sequencing (Figure 5b). At the *OsPDS*‐sgRNA01 site, mutation frequencies of T0 lines for STU‐Cas9‐RZ, STU‐Cas9‐Csy4 and STU‐Cas9‐tRNA were 79.5%, 90.6% and 86.7% respectively. At the *OsDEP1*‐sgRNA01 site, mutation frequencies of T0 lines for STU‐Cas9‐RZ, STU‐Cas9‐Csy4 and STU‐Cas9‐tRNA were 87.2%, 90.6% and 93.3% respectively. Importantly, majority of the mutants carried biallelic mutations at both target sites (Figure 5b). Second, we simultaneously targeted three sites: *OsPDS*‐sgRNA01, *OsPDS*‐sgRNA02 and *OsDEP1*‐sgRNA01 (Figure 6a). Again, more than 30 individual T0 plants were genotyped for each construct (Figure 6b). At the *OsPDS*‐sgRNA01 site, mutation frequencies of T0 lines for STU‐Cas9‐RZ, STU‐Cas9‐Csy4 and STU‐Cas9‐tRNA were 83.3%, 93.9% and 89.2% respectively. At the *OsPDS*‐sgRNA02 site, mutation frequencies of T0 lines for STU‐Cas9‐RZ, STU‐Cas9‐Csy4 and STU‐Cas9‐tRNA were 58.3%, 60.6% and 67.6% respectively. At *OsDEP1*‐sgRNA01 site, mutation frequencies of T0 lines for STU‐Cas9‐RZ, STU‐Cas9‐Csy4 and STU‐Cas9‐tRNA were 86.1%, 84.8% and 89.2% respectively. While *OsPDS*‐sgRNA02 showed relatively low editing activity, majority of the mutants still carried biallelic mutations (Figure 6b). Impressively, the STU‐Cas9‐tRNA system resulted highest triple biallelic knockout frequency: 23 out of 25 triple mutants carried biallelic mutations at all three target sites (Figure 6b).

### STU‐Cas9‐tRNA system for multiplexed editing of six target sites

**Figure 7:**
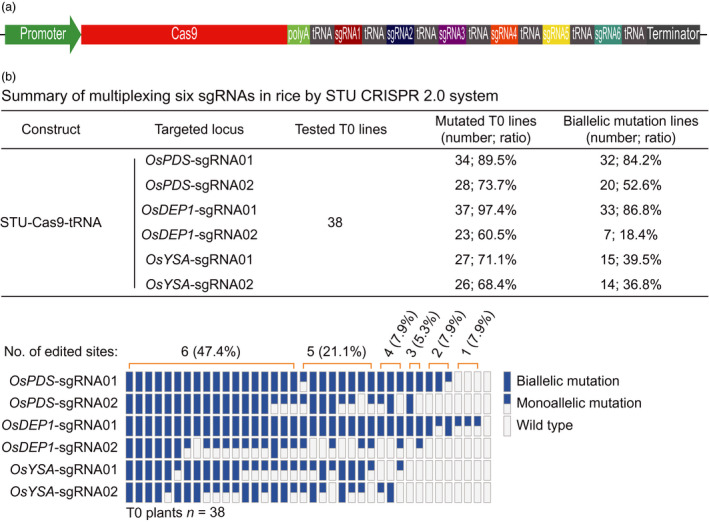
STU‐Cas9‐tRNA system with multiplexed six sgRNAs in rice transgenic lines. (a) Schematics of the STU‐Cas9‐tRNA expression construct. (b) Upper panel: a summary table of mutation frequencies at three target sites by the STU‐Cas9‐tRNA system; lower panel: a schematic presentation of the genotyping results for all T0 lines categorized as biallelic mutation, monoallelic mutation and wild type.

Having identified that STU‐Cas9‐Csy4 and STU‐Cas9‐tRNA are robust in genome editing, we were inclined to further explore STU‐Cas9‐tRNA, which is simpler than STU‐Cas9‐Csy4, for high capacity multiplexing. To this end, we multiplexed all the six chosen sgRNAs into a single STU‐Cas9‐tRNA T‐DNA construct (Figure 7a) and obtained 38 T0 transgenic lines for genotyping (Figure 7b). The mutation frequencies at *OsPDS*‐sgRNA01, *OsPDS*‐sgRNA02, *OsDEP1*‐sgRNA01, *OsDEP1*‐sgRNA02, *OsYSA*‐sgRNA01 and *OsYSA*‐sgRNA02 sites were 89.5%, 73.7%, 97.4%, 60.5%, 71.1% and 68.4% respectively. The editing efficiencies at *OsPDS*‐sgRNA01, *OsPDS*‐sgRNA02 and *OsDEP1*‐sgRNA01 sites were comparable to the results from the STU‐Cas9‐tRNA construct when only these three sgRNAs were multiplexed (Figure 6b), suggesting that high capacity multiplexing can be achieved without compromising editing efficiencies. Among 38 T0 lines, 18 lines (47.4%) contained mutations at all six target sites, and four lines carried biallelic mutations at each target site, indicating simultaneous mutagenesis at six sites are readily achievable with the STU‐Cas9‐tRNA system.

### Application of the STU‐Cas9 system for base editing

**Figure 8:**
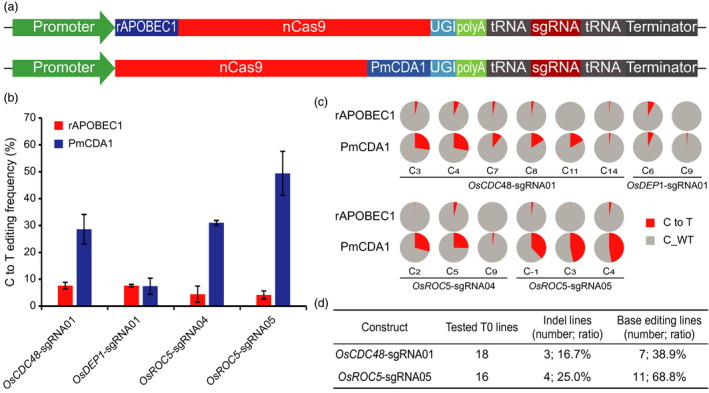
Comparison of two STU‐Cas9 base editing systems in rice. (a) Schematics of the two STU‐Cas9 base editing vectors for expressing one sgRNA each. (b) C to T base editing frequencies at four target sites. The editing frequencies were calculated by combining all the base changes within the target windows by analysing the deep sequencing data. Error bars represent standard deviations of two biological replicates. (c) Pie chart presentation of the frequencies of base changes at all the targetable cytosine sites by both STU‐Cas9 base editors at four target sites. Note the average frequencies of two biological replicates are shown in red. (d) Summary of genome editing in T0 lines at two target sites, *OsCDC48*‐sgRNA01 and *OsROC5*‐sgRNA05.

In recent years, both C to T and A to G base editing systems have been developed for inducing targeted base changes without DNA DSBs (Gaudelli *et al*., 2017; Komor *et al*., 2016). To test whether a STU‐Cas9 system can be used for base editing, we focused on comparison of two C to T base editing systems which are based on fusion of nCas9 (Cas9_D10A) with two different cytidine deaminases: rAPOBEC1 (Komor *et al*., 2016) and PmCDA1 (Shimatani *et al*., 2017). We constructed the STU‐nCas9‐tRNA to express these two base editors and compared them by targeting four independent sites in the rice genome (Figure 8a). The resulting T‐DNA constructs were used for transient transformation of rice protoplasts and the based editing efficiency, as sum of all targetable cytidines (Cs) within the targeting window for each target site, was calculated by deep sequencing. The results showed that PmCDA1 base editor significantly outperformed rAPOBEC1 base editor at three of the four target sites (Figure 8b). While the editing efficiencies by rAPOBEC1 were below 10%, the editing efficiencies by PmCDA1 were 30% and above for *OsCDC48*‐sgRNA01, *OsROC5*‐sgRNA04 and *OsROC5*‐sgRNA05 (Figure 8b). A detailed analysis on individual targetable Cs within the base editing windows across these sites consistently pointed to much higher C to T conversion rates with the PmCDA1 base editor system (Figures 8c and S2).

To obtain base‐edited plants, we transformed two T‐DNA constructs with *OsCDC48*‐sgRNA01 and *OsROC5*‐sgRNA05 into rice to generate stable transgenic lines. Eighteen T0 lines for each construct were screened for editing at the target site. For *OsCDC48*‐sgRNA01, seven out of 18 lines (38.9%) carried C to T base changes, while three lines contained deletions at the target site (Figures 8c and S3). For *OsROC5*‐sgRNA05, 11 out of 18 lines (68.8%) carried C to T base changes, while four lines contained deletions at the target site (Figures 8c and S4). These results suggest the STU‐nCas9‐PmCDA1 base editing system is very efficient in generating plants with targeted C to T base changes.

### A STU‐Cas12a system for multiplexed genome editing

**Figure 9:**
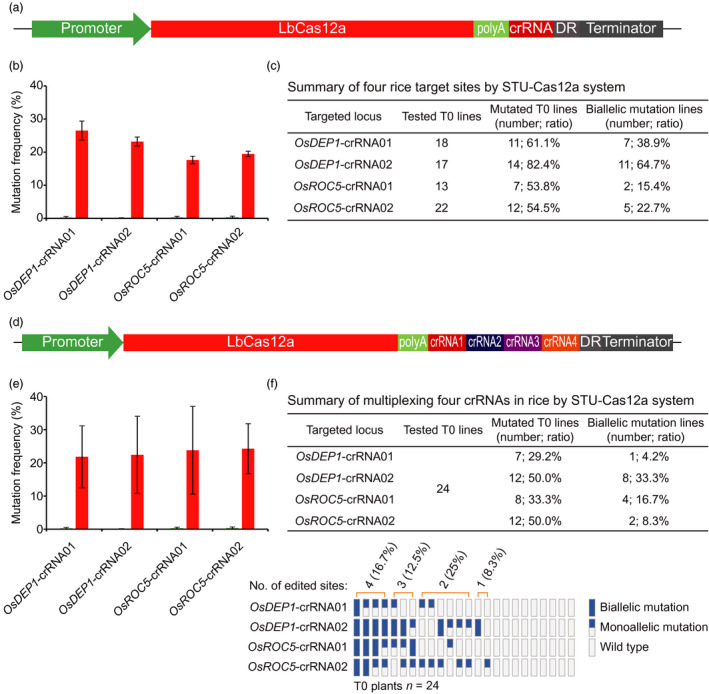
A STU‐Cas12a system for plant genome editing. (a) Schematics of the STU‐Cas12a system expressing one crRNA, which is flanked by direct repeats (DRs) for processing by Cas12a. (b) Mutation frequencies at four target sites by the STU‐Cas12a system. The experiments were carried out in rice protoplasts and the frequencies were measured by amplicon‐based deep sequencing. Error bars represent standard deviations of two biological replicates. (c) Mutation frequencies at four target sites by the STU‐Cas12a system. (d) Schematics of the STU‐Cas12a system expressing four crRNAs, which are flanked by DRs for processing by Cas12a. (e) Mutation frequencies at four target sites by the multiplexed STU‐Cas12a system. The experiments were carried out in rice protoplasts and the frequencies were measured by amplicon‐based deep sequencing. Error bars represent standard deviations of two biological replicates. (f) Upper panel: a summary table for mutation frequencies at four target sites; lower panel: a schematic presentation of the genotyping results for all T0 lines categorized as biallelic mutation, monoallelic mutation and wild type.

We also sought to demonstrate that CRISPR‐Cas12a can be expressed as a STU. Self‐processing of a crRNA array has been successfully used for multiplexed editing by Cas12a in human cells (Zetsche *et al*., 2017) and in plants (Wang *et al*., 2017b). It has been shown that the addition of an extra direct repeat (DR) at the end of the crRNA array helped process the last crRNA and hence boosted its activity (Zhong *et al*., 2017). Among all three Cas12a nucleases (AsCas12a, LbCas12a and FnCas12a) tested in plants, LbCas12a seems to have robust activity (Hu *et al*., 2017; Tang *et al*., 2017; Wang *et al*., 2017b; Xu *et al*., 2016; Zhong *et al*., 2018). We hence built an STU‐Cas12a system in which LbCas12a and the crRNA array were driven by a single Pol II promoter (pZmUbi) and separated by poly A (Figure 9a). With this system, we targeted four independent sites in the rice genome. An average of ~20% mutation frequencies were achieved across these sites in protoplasts (Figures 9b and S5). We next used these four constructs to generate stable transgenic T0 lines and genotyped them by CAPS analysis (Figure S6) and Sanger sequencing. At *OsDEP1*‐crRNA01, *OsDEP1*‐crRNA02, *OsROC5*‐crRNA01 and *OsROC5*‐crRNA02, mutation frequencies of T0 lines were 61.1%, 82.4%, 53.8% and 54.5% respectively; biallelic mutation frequencies were 38.9%, 64.7%, 15.4% and 22.7% respectively (Figure 9c). We next generated a multiplexed STU‐Cas12a construct targeting four crRNAs simultaneously (Figure 9d). Using a protoplast assay (Figure S7), we found an average of ~20% mutation frequencies at all four target sites (Figure 9e). This construct was further used to generate T0 transgenic lines followed by CAPS analysis (Figure S8) and Sanger sequencing. Analysis of 24 T0 lines revealed mutation frequencies of 29.2%, 50%, 33.3% and 50% at *OsDEP1*‐crRNA01, *OsDEP1*‐crRNA02, *OsROC5*‐crRNA01 and *OsROC5*‐crRNA02 respectively (Figure 9f). Biallelic mutations were identified for each crRNA at variant efficiencies and one T0 line contained biallelic mutations at all four target sites (Figure 9f).

## Discussion

In a previous review, we proposed three promising STU‐Cas9 systems to be tested and deployed for plant genome editing, which are based on ribozyme, Csy4 and tRNA respectively (Lowder *et al*., 2016). We demonstrated STU‐Cas9‐RZ system in plants earlier (Tang *et al*., 2016). In this study, we developed the STU‐Cas9‐Csy4 and STU‐Cas9‐tRNA systems and closely compared them with STU‐Cas9‐RZ and the conventional mixed dual promoter system. Our data suggest the STU‐Cas9‐RZ system had similar editing efficiency with the conventional mixed dual promoter system (Figure 1), which is consistent with our previous report (Tang *et al*., 2016). However, we found the new STU‐Cas9‐Csy4 and STU‐Cas9‐tRNA systems showed more robust genome editing as compared to STU‐Cas9‐RZ (Figures 1, 5 and 6). Hence, these two STU‐Cas9 2.0 systems represent improved STU‐Cas9 expression systems for plant applications. Our results were consistent with a previous study, which used a dual Pol II promoter system and showed that Cas9‐Csy4 and Cas9‐tRNA systems had higher editing efficiencies than the Cas9‐RZ system (Cermak *et al*., 2017). We however want to note that in these RZ systems the sgRNA is flanked by two HH ribozyme cleavage sites with only one full HH ribozyme sequence at the 3′ end, which thus requires trans‐cleavage activity of the HH ribozyme. By contrast, the HH ribozyme was positioned at the 5′ end of a guide RNA for self‐cleavage in the dual ribozyme system that we used for the development of a highly efficient Cas12a system (Tang *et al*., 2017; Zhong *et al*., 2018).

The use of deep sequencing allowed us to thoroughly compare NHEJ repair outcomes of the four Cas9 expression systems across six different target sites. As anticipated, our results suggest Cas9 expression systems themselves did not affect the editing outcomes as the mutation types and frequencies across four expression systems were strikingly similar (Figures 2 and 3). Hence, the four different Cas9 expression systems rather served as additional biological replicates for studying NHEJ repair outcomes. We found the repair outcomes are heavily dependent of target site sequences. For example, the mutation profiles at the six target sites are all different from each other, in terms of deletion positions and sizes (Figures 2 and 3). This observation indicates the frequent involvement of the altNHEJ pathway which depends on sequence microhomology. Previously, we showed microhomology‐based altNHEJ in repair of zinc finger nuclease (ZFN) induced DNA DSBs in *Arabidopsis* and the use of altNHEJ was drastically boosted when the Ku70/Ku80 based canonical NHEJ pathway was blocked (Qi *et al*., 2013). Our study here, however, suggests that microhomology‐based altNHEJ seems to be more frequent than previously thought in repair of Cas9‐generated DNA DSBs in wild‐type rice cells. Microhomology‐based altNHEJ has been cleverly used for precise genome editing in human cells, given the editing outcomes were partly predictable (Bae *et al*., 2014). Since HDR frequency is low in plants, it is appealing to use altNHEJ to introduce desired deletions at target sites in plants.

While we were preparing this manuscript, three groups have published additional STU‐Cas9 systems in rice (Ding *et al*., 2018; Mikami *et al*., 2017; Wang *et al*., 2018a). Mikami *et al*. made a striking finding that sgRNAs, when directly fused to the Cas9 coding sequence, can be processed by unknown ribonucleases in rice to result in functional CRISPR‐Cas9 ribonuclear protein complex (Mikami *et al*., 2017). They showed multiplexed genome editing by expressing two sgRNAs. Inspired by this research, the second study by Wang *et al*. developed a simplified STU‐Cas9 system in which the sgRNAs were separated by 6‐bp linkers (Wang *et al*., 2018a). The third study by Ding *et al*. used the intron of the Cas9 cassette to express one or a few sgRNAs (Ding *et al*., 2018), an idea similar to the one previously demonstrated in Chlamydomonas (Jiang and Weeks, 2017). It is hard for us to make direct comparison between our STU‐Cas9 2.0 systems and the STU‐Cas9 system developed by Mikami *et al*., since they only genotyped rice calli, not independent T0 plants (Mikami *et al*., 2017). However, the first two studies used a similar STU strategy that relies on *in planta* processing of sgRNAs (Mikami *et al*., 2017; Wang *et al*., 2018a). We compared the work by Wang *et al*. with ours as both studies generated T0 transgenic lines for analysis. Wang *et al*. targeted three sites for multiplexed editing with their simple STU‐Cas9 system. While the mutation frequencies at the three sites in T0 lines are relatively comparable to our study, none of their analysed T0 lines contained biallelic mutations at all three target sites at once. Also, chimeric lines were found in some cases (Wang *et al*., 2018a). Similarly, Ding *et al*. achieved simultaneous biallelic mutation rates of 9.7% (3/31) with PTG‐6 and 0% (0/8) with PTG‐7 when two sgRNAs and four sgRNAs were multiplexed in their intron‐based STU vectors even though the tRNA processing system was also used (Ding *et al*., 2018). By contrast, our STU‐Cas9 2.0 systems resulted in biallelic mutations at high frequencies at all targeted sites among the T0 lines. For example, when two sgRNAs were multiplexed with STU‐Cas9‐Csy4 and STU‐Cas9‐tRNA, we observed simultaneous biallelic mutation frequencies of 75% (24/32) and 73.3% (22/30) respectively (Figure 5). When three sgRNAs were multiplexed with STU‐Cas9‐Csy4 and STU‐Cas9‐tRNA, we found simultaneous biallelic mutation frequencies of 39.4% (13/33) and 62.2% (23/37) (Figure 6). While none of the three published studies have expressed more than four sgRNAs with their STU‐Cas9 systems, we multiplexed six target sites with STU‐Cas9‐tRNA and could readily identified T0 lines (4/38; 10.5%) in which all six target sites contain biallelic mutations (Figure 7). Hence, our new STU‐Cas9 2.0 systems showed highest editing efficiency especially in terms of achieving biallelic knockouts. Furthermore, Csy4 and tRNA‐based processing systems have been demonstrated with high editing activities in plants and mammalian systems (Cermak *et al*., 2017; Port and Bullock, 2016; Qin *et al*., 2015; Shiraki and Kawakami, 2018; Tsai *et al*., 2014; Xie *et al*., 2015). It is conceivable that our STU‐Cas9‐Csy4 and STU‐Cas9‐tRNA are likely to work well in other organisms. In the future, it will be interesting to compare these STU‐Cas9 systems with other STU‐Cas9 systems (Ding *et al*., 2018; Mikami *et al*., 2017; Wang *et al*., 2018a) in many other plant species like dicots.

CRISPR‐Cas9 can be converted to nickases for promoting targeting specificity (Fauser *et al*., 2014; Ran *et al*., 2013), stimulating HDR (Cermak *et al*., 2015; Miki *et al*., 2018; Sun *et al*., 2016; Wang *et al*., 2017a), and repurposing for base editing (Gaudelli *et al*., 2017; Komor *et al*., 2016; Shimatani *et al*., 2017). Furthermore, deactivated Cas9 (dCas9) has been used for engineering synthetic transcriptional regulators (Lowder *et al*., 2015, 2018; Piatek *et al*., 2014; Tang *et al*., 2017) and DNA labelling in plants (Dreissig *et al*., 2017). It is conceivable that our STU‐Cas9 2.0 systems can be applied in all these applications. As a proof‐of‐concept, we applied the STU‐Cas9‐tRNA system to express two C to T base editing systems in rice. Our data suggest the STU‐nCas9‐PmCDA1 base editing system is highly efficient, resulting in up to 50% base editing frequencies in its target window when analysed in rice protoplasts (Figure 8). While the rAPOBEC1 base editor is frequently used in plants (Lu and Zhu, 2017; Zong *et al*., 2017), PmCDA1 base editor resulted in much higher base editing efficiencies in our study. However, we are cautioned to claim that PmCDA1 base editor is inherently more efficient than rAPOBEC1 base editor in plants, because the low activity of rAPOBEC1 base editor could be due to suboptimal codon optimization of the rAPOBEC1 sequence. A recent study in human cells suggest codon optimization can significantly improve base editing efficiency (Koblan *et al*., 2018). Nevertheless, we have developed a highly efficient STU‐Cas9‐tRNA based PmCDA1 base editor system for base editing in plants. In the future, it will be interesting to compare PmCDA1 with human APOBEC3A as the latter was recently shown to have high C to T base editing activities in plants (Zong *et al*., 2018) and can efficiently target methylated regions with minimized bystander and off‐target activities in mammalian cells (Gehrke *et al*., 2018; Wang *et al*., 2018b). We also observed indels in T0 plants transformed with the STU‐nCas9‐PmCDA1 base editor, and the relatively high frequency of indel mutations (~20% on average) is consistent with the previous reports of the PmCDA1 base editor (Shimatani *et al*., 2017) as well as the rAPOBEC1 base editor (Li *et al*., 2017; Lu and Zhu, 2017). Such deletions were likely resulted from NHEJ repair of chromosomal nicks in plant cells. Future efforts should also be directed to minimize such unintended NHEJ outcomes without compromising base editing efficiency.

In this study, we also developed a STU‐Cas12a system and compared it for expressing one and four crRNAs in rice for genome editing. We found editing frequencies were slightly dropped when the same crRNA was moved into a crRNA array for multiplexed editing. The two recent reports also explored STU‐Cas12a systems (Ding *et al*., 2018; Wang *et al*., 2018a). Ding *et al*. expressed a crRNA array with an intron to multiplex two crRNAs for targeted gene deletion (Ding *et al*., 2018). However, the study was done in rice protoplasts and no stable transformation data were available for comparison with ours. Wang *et al*. reported a STU‐Cas12a system in which they flanked the crRNA array with two tRNA sequences (Wang *et al*., 2018a). They found their STU‐Cas12a system resulted in similar editing frequencies when multiplexing nine crRNAs, and the editing frequencies ranged from 4.2% to 70.8% in rice T0 lines (Wang *et al*., 2018a). Given LbCas12a can self‐process a crRNA array, we did not flank our crRNA array with tRNA sequences. Otherwise, the two STU‐Cas9 strategies are similar as both link the LbCas12a and the crRNA array together under a single Pol II promoter. We achieved editing efficiencies from 29.2% to 50% at the four target sites, within a similar range as their work (Wang *et al*., 2018a). With a dual Pol II promoter and a double ribozyme system, we previously showed very high biallelic mutation rates in rice with LbCas12a (Tang *et al*., 2017) and FnCas12a (Zhong *et al*., 2018). Given all the STU‐Cas12a systems reported to date are all based on the self‐processing of crRNA arrays by Cas12a, in the future, it may be worthwhile to test other multiplexing strategies to further improve Cas12a mediated genome editing in plants.

## Conclusion

In conclusion, we developed two STU‐Cas9 2.0 systems that process sgRNAs with Csy4 and tRNA. The STU‐Cas9 2.0 systems displayed more robust genome editing activities when compared to our first‐generation STU‐Cas9‐RZ system and the conventional mixed dual promoter system. We achieved high‐frequency base editing using the new STU‐Cas9‐tRNA system with the PmCDA1 base editor. We also developed a STU‐Cas12a system for multiplexed plant genome editing. While the study was done in rice, we anticipate the systems that we developed here will have wide applications for expressing CRISPR systems in diverse plant species.

## Experimental procedures

### Construction of the vectors

The Cas9 vectors of this study were constructed based on the STU Cas9 vector pTX172 in our previous study (Tang *et al*., 2016). To construct the STU‐Cas9‐RZ system backbone (pGEL029), the Hsp terminator was amplified from pZHY988B and then cloned into the *Sac*I restriction site of pTX172. To construct the STU‐Cas9‐Csy4 system backbone (pGEL030), the Csy4‐P2A fragment was amplified from pMOD_A0501 (Cermak *et al*., 2017) and the Cas9‐polyA fragment and the Csy4 site‐ccdB‐gRNA‐Csy4 site fragment were amplified from pTX172. The above three fragments were cloned into *Sbf*I + *Hind*III linearized STU‐Cas9‐RZ plasmids by Gibson assembly. To generate the STU‐Cas9‐tRNA system backbone (pGEL031), the Cas9‐polyA fragment was amplified from pTX172 and the tRNA‐ccdB‐gRNA‐tRNA fragment was amplified from pTC315. These two fragments were cloned into *Sbf*I + *Hind*III linearized STU‐Cas9‐RZ plasmids by Gibson assembly. For single gene editing, sgRNAs were synthesized as duplexed oligonucleotides (Table S1). Oligos were annealed and cloned into *Bsa*I linearized Cas9 vectors. For multiplex gene editing, sgRNA arrays were generated by PCR and cloned into the Cas9 vectors by Golden Gate cloning (Table S1).

The Cas9 base editing constructs were constructed based on the STU‐Cas9‐tRNA system. To construct rAPOBEC1 base editing system backbone (pGEL033), rAPOBEC1 fragment, nCas9 fragment and UGI fragment were amplified separately, and then cloned into the STU‐Cas9‐tRNA system by Gibson Assembly. To construct PmCDA1 base editing system backbone (pGEL035), nCas9 fragment and PmCDA1‐UGI fragment were amplified and cloned into the STU‐Cas9‐tRNA system by Gibson Assembly.

To construct the STU Cas12a system backbone (pGEL032), the LbCas12a fragment was amplified from pYPQ230 (Tang *et al*., 2017) and the polyA‐ccdB‐DR fragment was synthesized by Genscript Nanjing Ltd. These two fragments were cloned into *Sbf*I + *Hind*III linearized STU‐Cas9‐RZ plasmids by Gibson assembly. For single gene editing, crRNAs were synthesized as duplexed oligonucleotides (Table S1). Oligos were annealed and cloned into *Bsa*I linearized STU Cas12a vectors. For multiplex gene editing, crRNA arrays were generated by overlap extension PCR and cloned into the STU Cas12a vectors by Golden Gate cloning (Table S1).

The plasmid maps of all the backbone vectors used in this study were provided as Supplemental files (pGEL029_2018, PBJ.gbk/pGEL030_2018, PBJ.gbk/pGEL031_2018, PBJ.gbk/pGEL032_2018, PBJ.gbk/pGEL033_2018, PBJ.gbk/pGEL035_2018, PBJ.gbk).

### Rice protoplast transformation and stable transformation

The *Oryza sativa* Geng/Japonica cultivar Nipponbare was used in this study. Rice protoplast transformation was performed as described previously (Tang *et al*., 2016; Zhang *et al*., 2013; Zhong *et al*., 2018). After transformation, rice protoplasts were incubated at 28 °C for 2 days before DNA extraction. The T‐DNA constructs were introduced into *Agrobacterium* EHA105 by the freeze‐thaw method. Rice stable transformation was carried out as previously published protocol (Tang *et al*., 2016; Zheng *et al*., 2016; Zhou *et al*., 2018).

### Detection of targeted gene mutations

Genomic DNA was extracted from protoplasts or transgenic plants by the CTAB method (Murray and Thompson, 1980). Genomic regions of targeted sites were amplified with specific primers (Table S1). Then the PCR products were digested by corresponding restriction enzymes overnight for CAPS analysis (Zhang *et al*., 2013). The digested products were analysed on 1% agarose gels. T0 mutant lines were further genotyped by Sanger sequencing.

### High‐throughput sequencing analysis

High‐throughput sequencing analysis was carried out as published previously (Tang *et al*., 2017; Zhong *et al*., 2018). Genome regions of targeted sites were PCR‐amplified using barcoded primers (Table S1). Purified DNA samples were quantified by Qubit 2.0 Fluorometer (Life Technologies, Waltham, MA) and were sequenced using Illumina Hiseq 2500 platform. Data processing was carried out using CRISPRMatch (You *et al*., 2018).

## Conflict of interest

The authors declare no competing interests.

## Supporting information


**Figure S1** CAPS analysis of STU‐Cas9 systems.
**Figure S2** Base editing efficiency at four target sites by rAPOBEC1 and PmCDA1 base editors.
**Figure S3** Detection of base editing in T0 rice lines by STU‐nCas9‐PmCDA1 with *OsCDC48*‐sgRNA01.
**Figure S4** Detection of base editing in T0 rice lines by STU‐nCas9‐PmCDA1 with *OsROC5*‐sgRNA05.
**Figure S5** CAPS analysis of the STU‐Cas12a system.
**Figure S6** CAPS analysis of T0 lines generated by the STU‐Cas12a system.
**Figure S7** Analysis of transiently transformed rice protoplasts for multiplexing four crRNAs using the STU‐Cas12a system.
**Figure S8** CAPS analysis of T0 lines generated by the multiplexed STU‐Cas12a system.

**Table S1** Oligos used in this study

**Supplemental files** The plasmid maps of all the backbone vectors used in this study

## Data Availability

The raw data of deep sequencing have been deposited to the Sequence Read Archive in National Center for Biotechnology Information (NCBI) under the accession number PRJNA49 4622.
