# Supplementary material for: Single transcript unit CRISPR 2.0 systems for robust Cas9 and Cas12a mediated plant genome editing
Source: Plant Biotechnol J. 2019 Jan 17;17(7):1431–45. doi: 10.1111/pbi.13068 (PMC6576101; doi:10.1111/pbi.13068)
Supplement: Supplementary file 1 — Figure S1 CAPS analysis of STU‐Cas9 systems. Figure S2 Base editing efficiency at four target sites by rAPOBEC1 and PmCDA1 base editors. Figure S3 Detection of base editing in T0 rice lines by STU‐nCas9‐PmCDA1 with OsCDC48‐sgRNA01. Figure S4 Detection of base editing in T0 rice lines by STU‐nCas9‐PmCDA1 with OsROC5‐sgRNA05. Figure S5 CAPS analysis of the STU‐Cas12a system. Figure S6 CAPS analysis of T0 lines generated by the STU‐Cas12a system. Figure S7 Analysis of transiently transformed rice protoplasts for multiplexing four crRNAs using the STU‐Cas12a system. Figure S8 CAPS analysis of T0 lines generated by the multiplexed STU‐Cas12a system. [file PBI-17-1431-s001.pptx]

## Slide 1
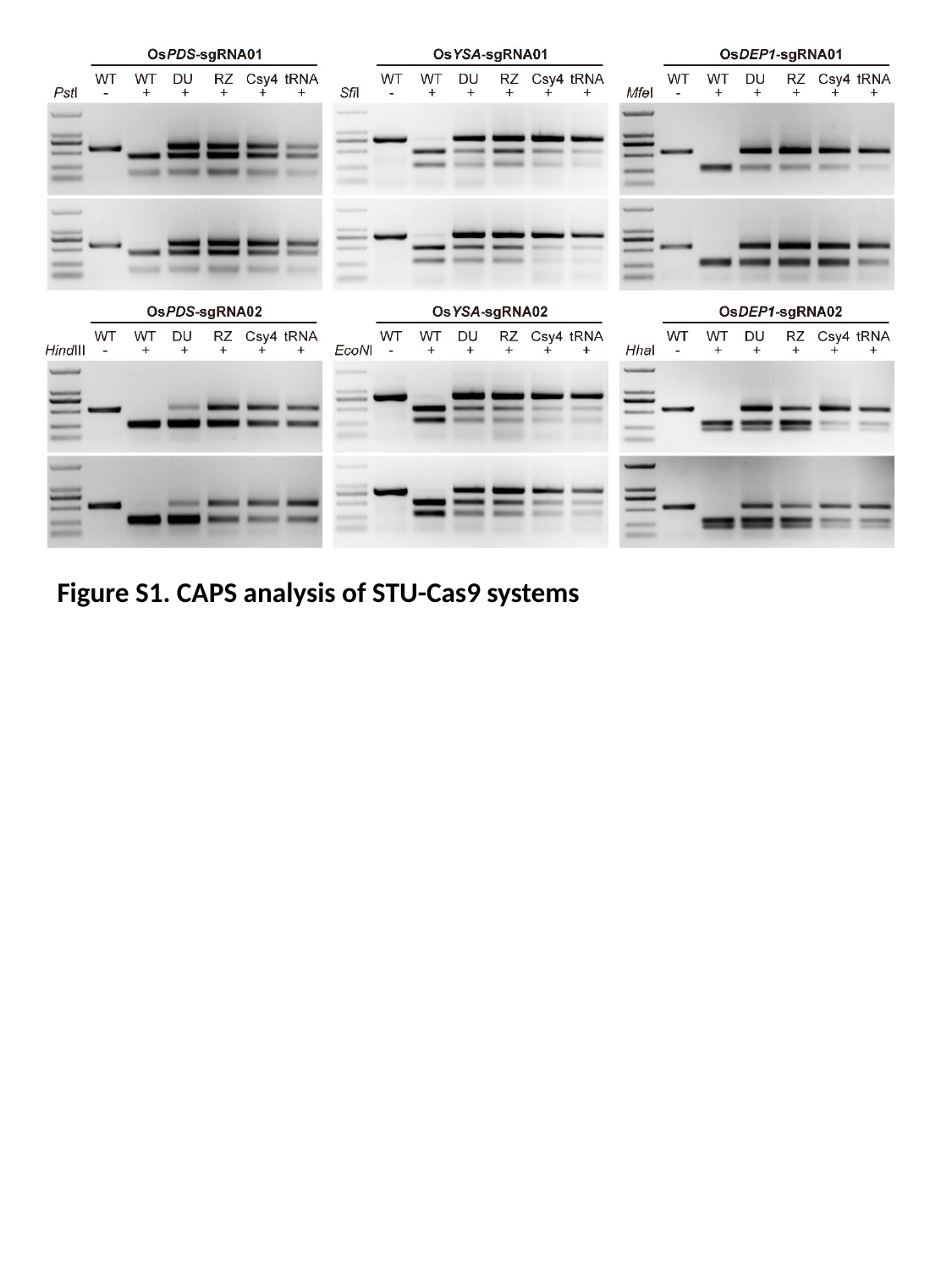

Figure S1. CAPS analysis of STU-Cas9 systems

## Slide 2
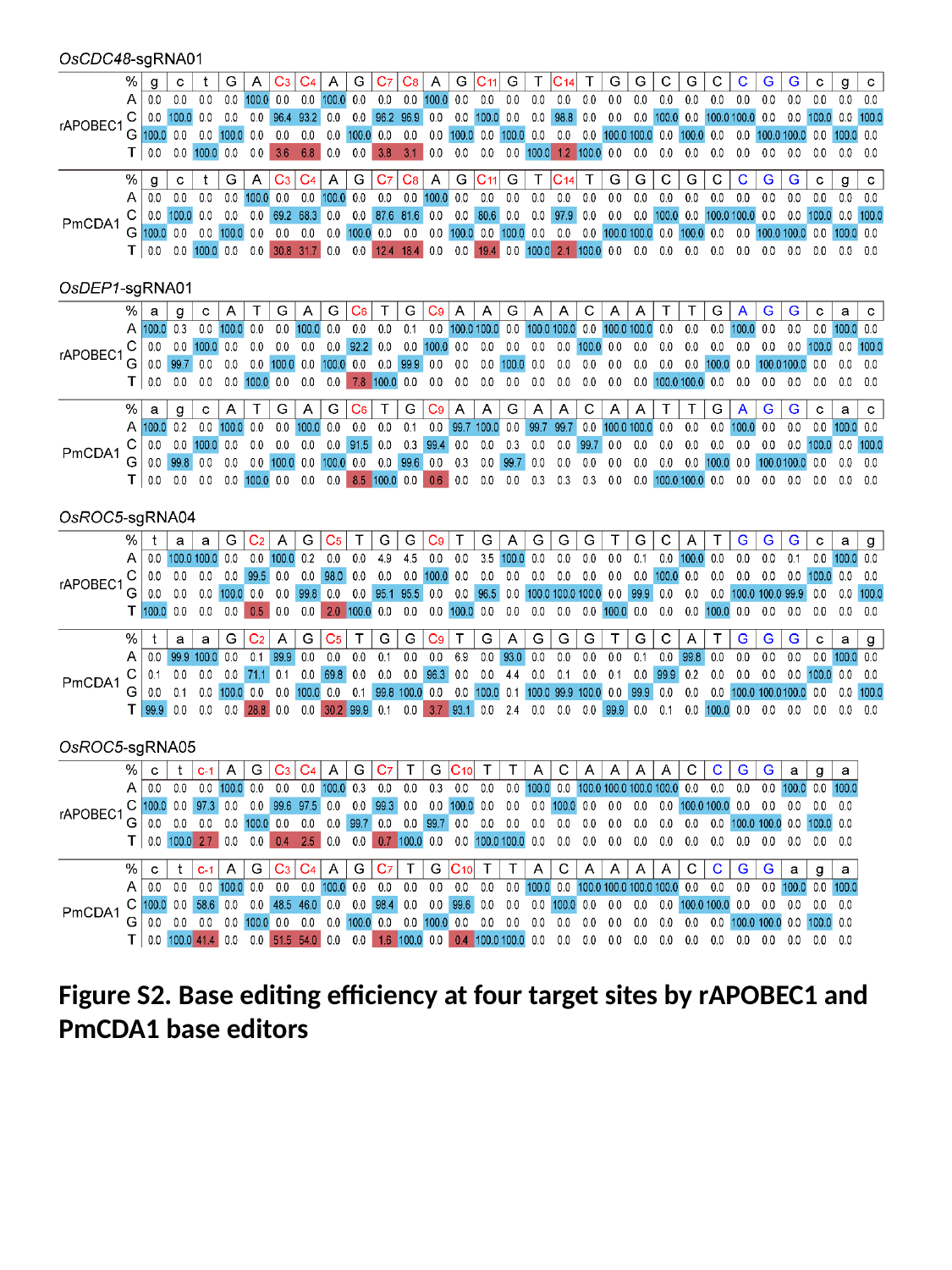

Figure S2. Base editing efficiency at four target sites by rAPOBEC1 and PmCDA1 base editors

## Slide 3
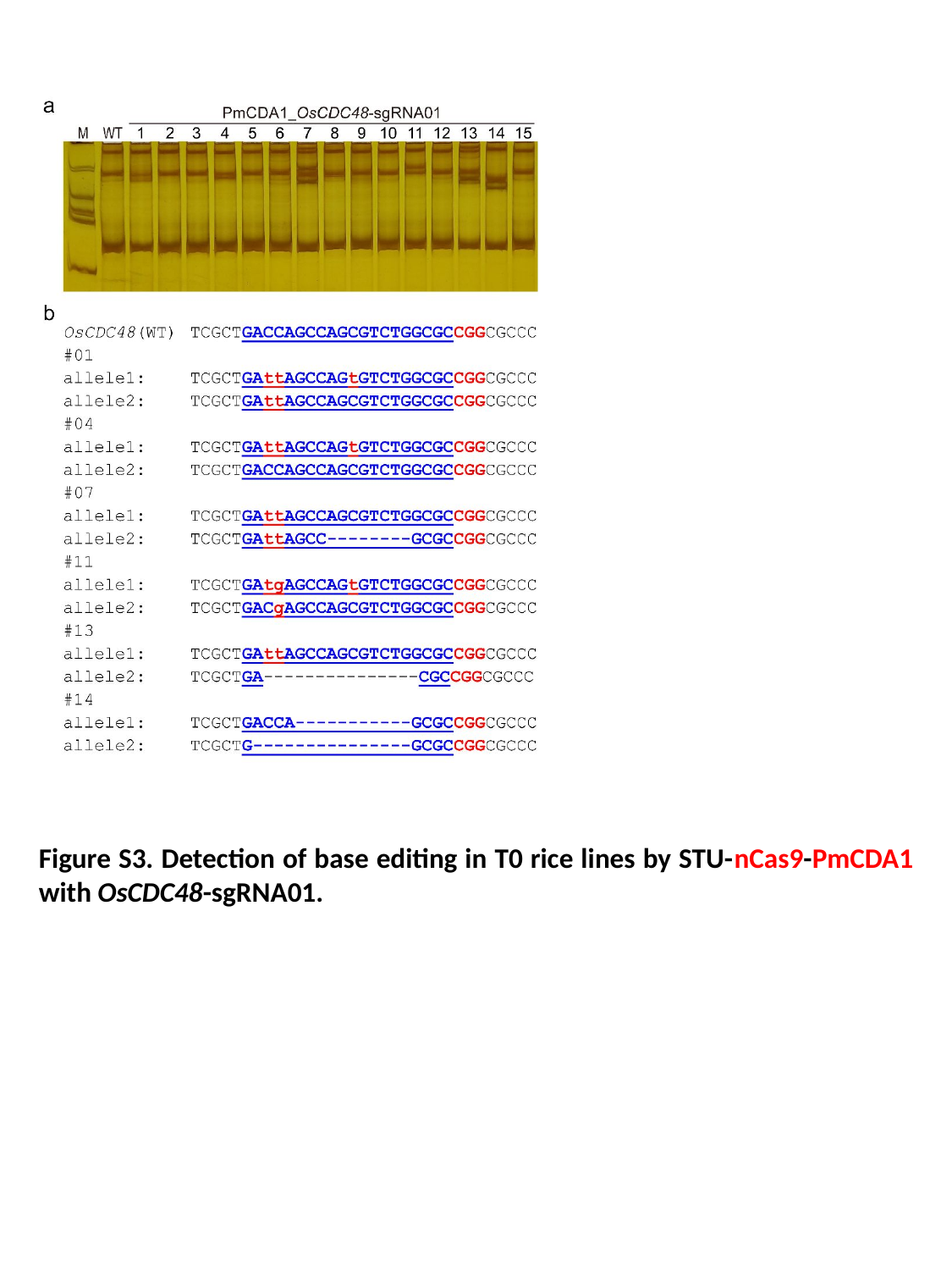

Figure S3. Detection of base editing in T0 rice lines by STU-nCas9-PmCDA1 with OsCDC48-sgRNA01.

## Slide 4
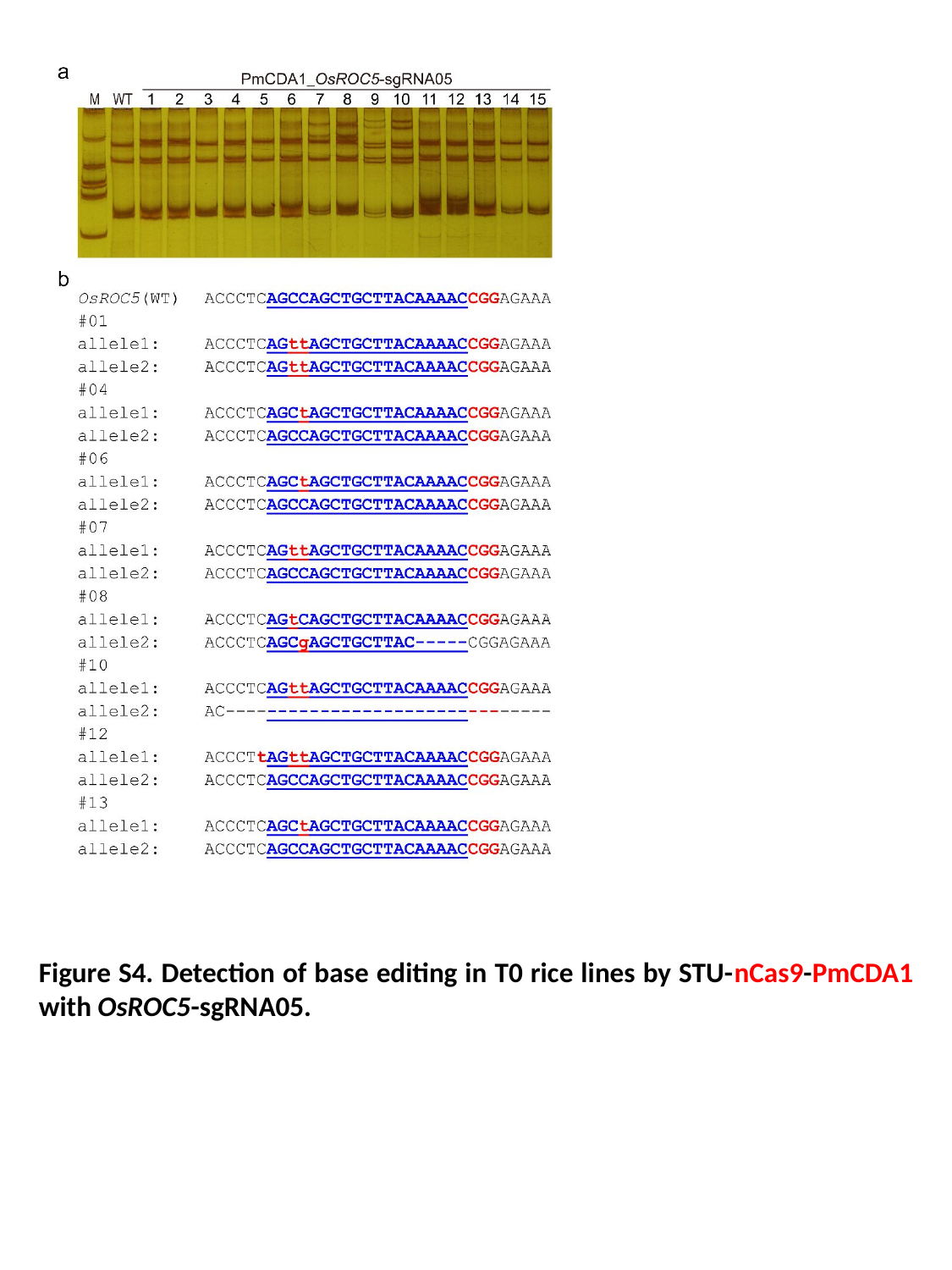

Figure S4. Detection of base editing in T0 rice lines by STU-nCas9-PmCDA1 with OsROC5-sgRNA05.

## Slide 5
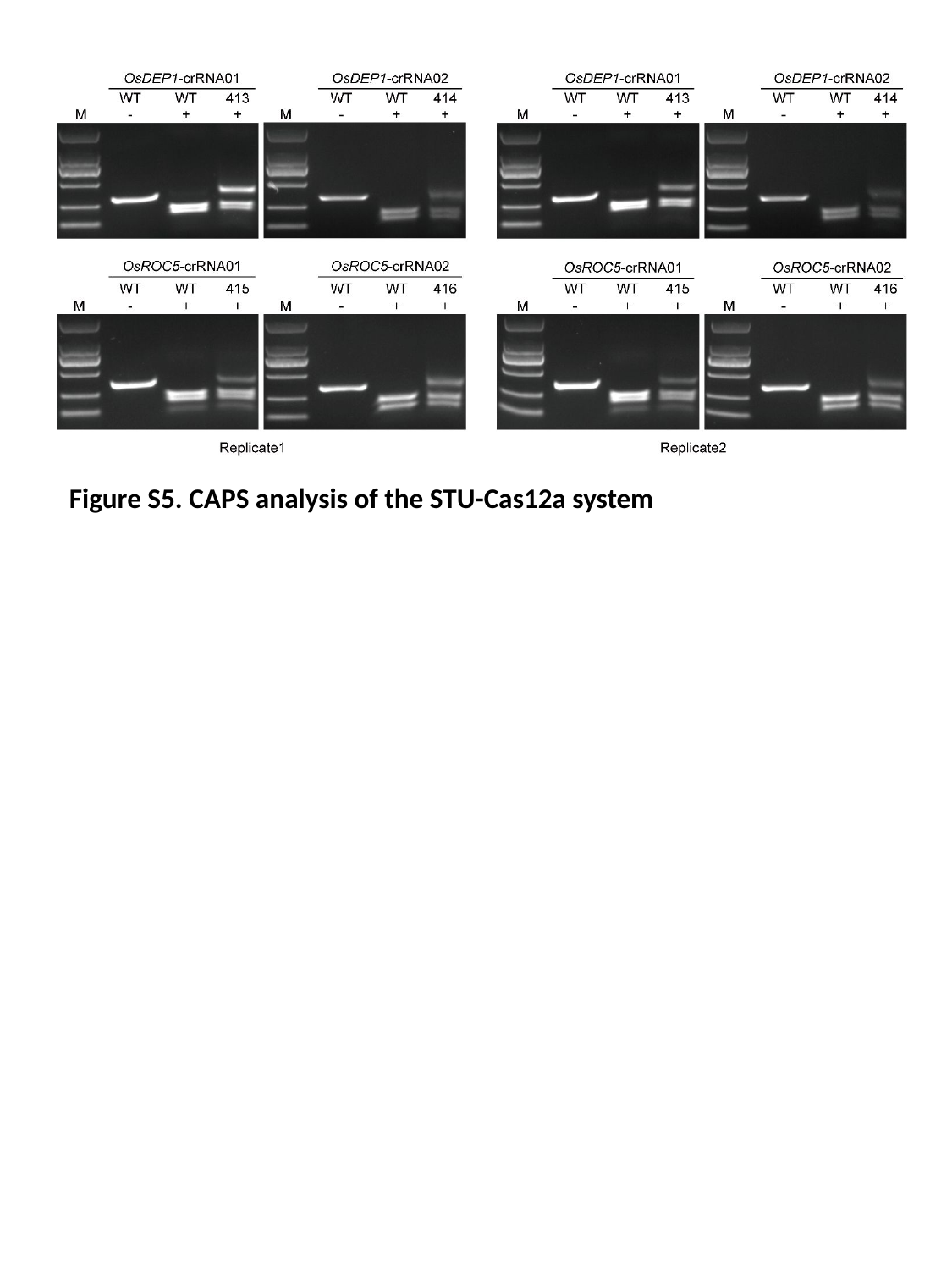

Figure S5. CAPS analysis of the STU-Cas12a system

## Slide 6
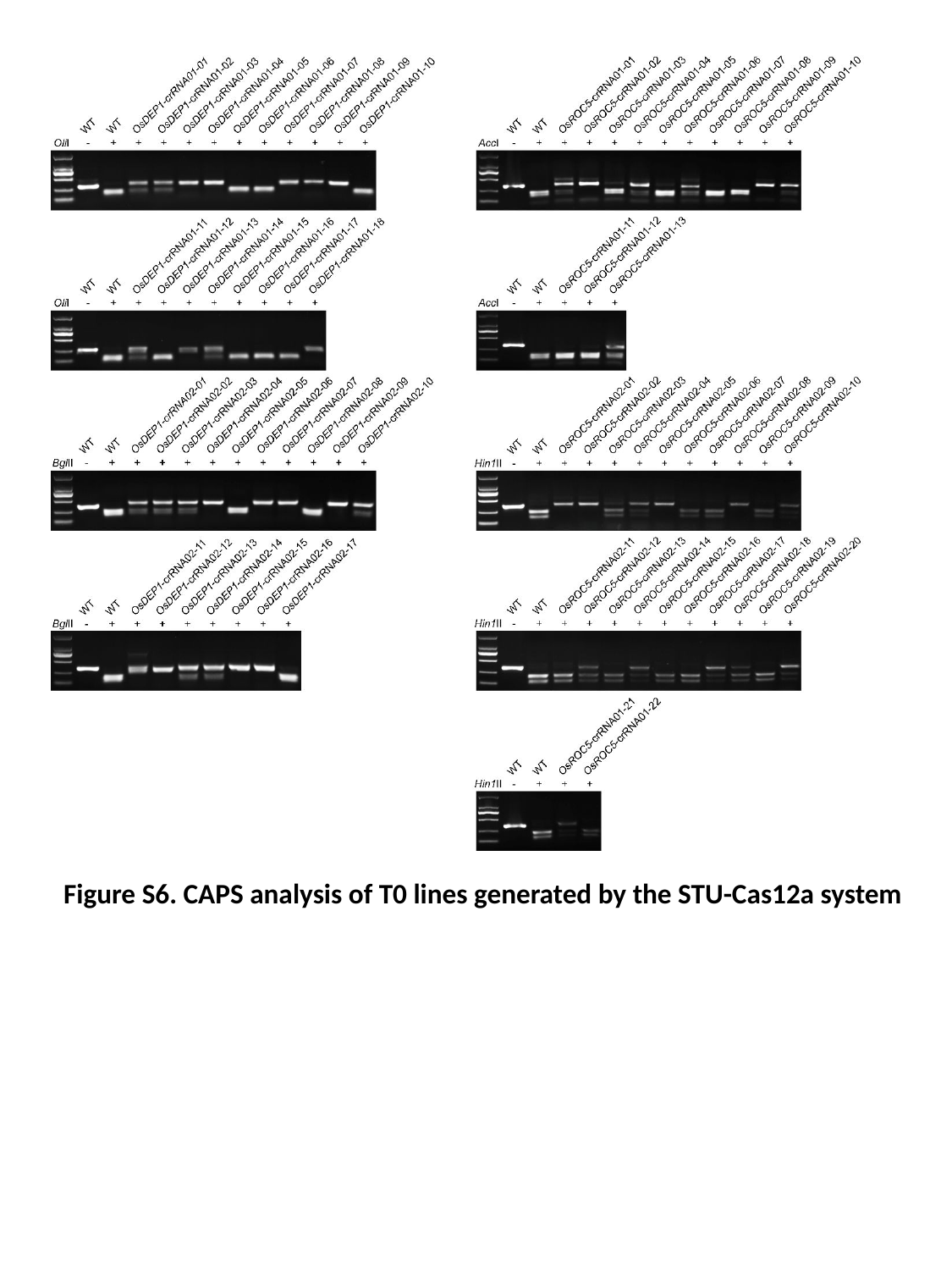

Figure S6. CAPS analysis of T0 lines generated by the STU-Cas12a system

## Slide 7
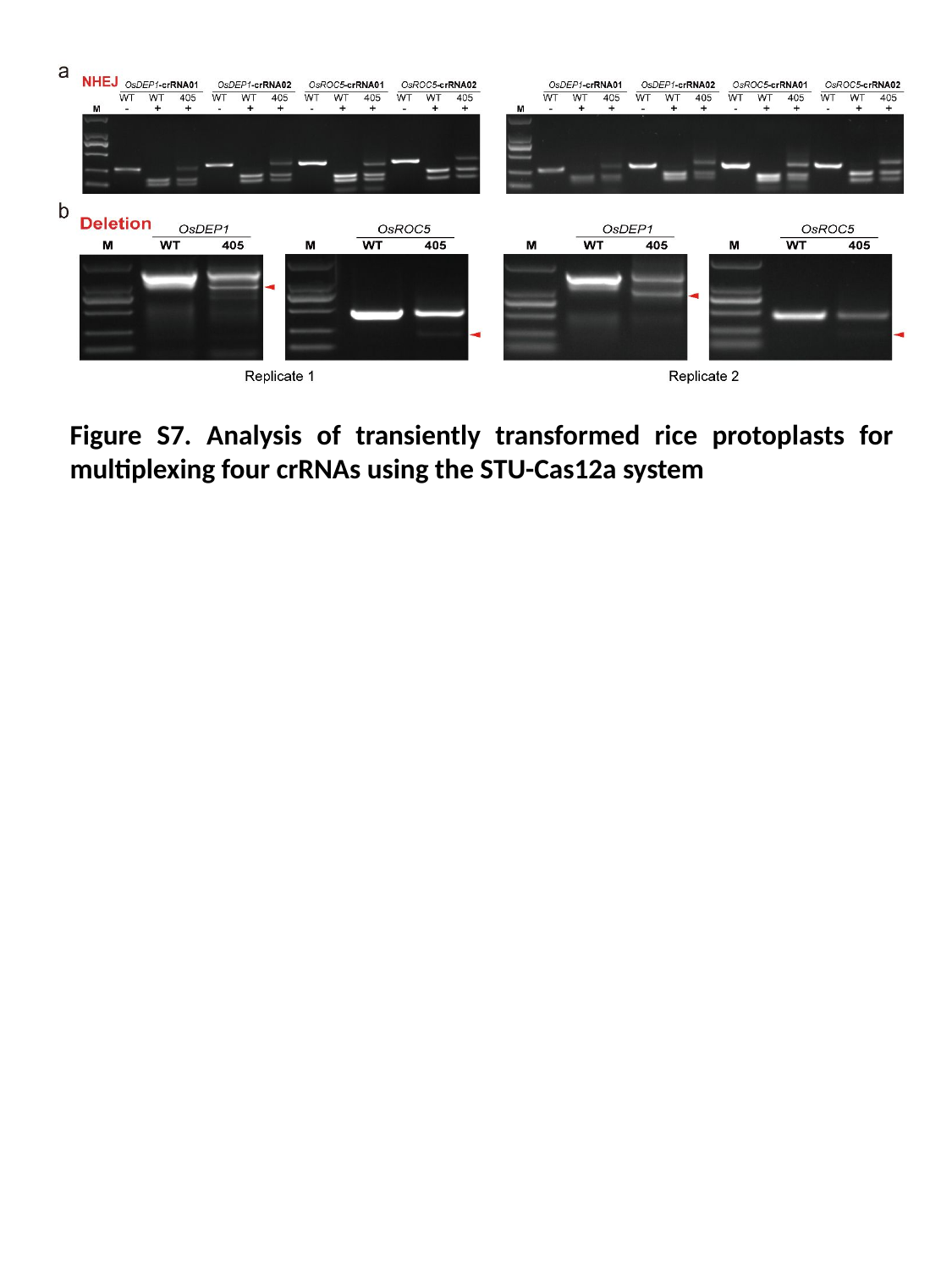

Figure S7. Analysis of transiently transformed rice protoplasts for multiplexing four crRNAs using the STU-Cas12a system

## Slide 8
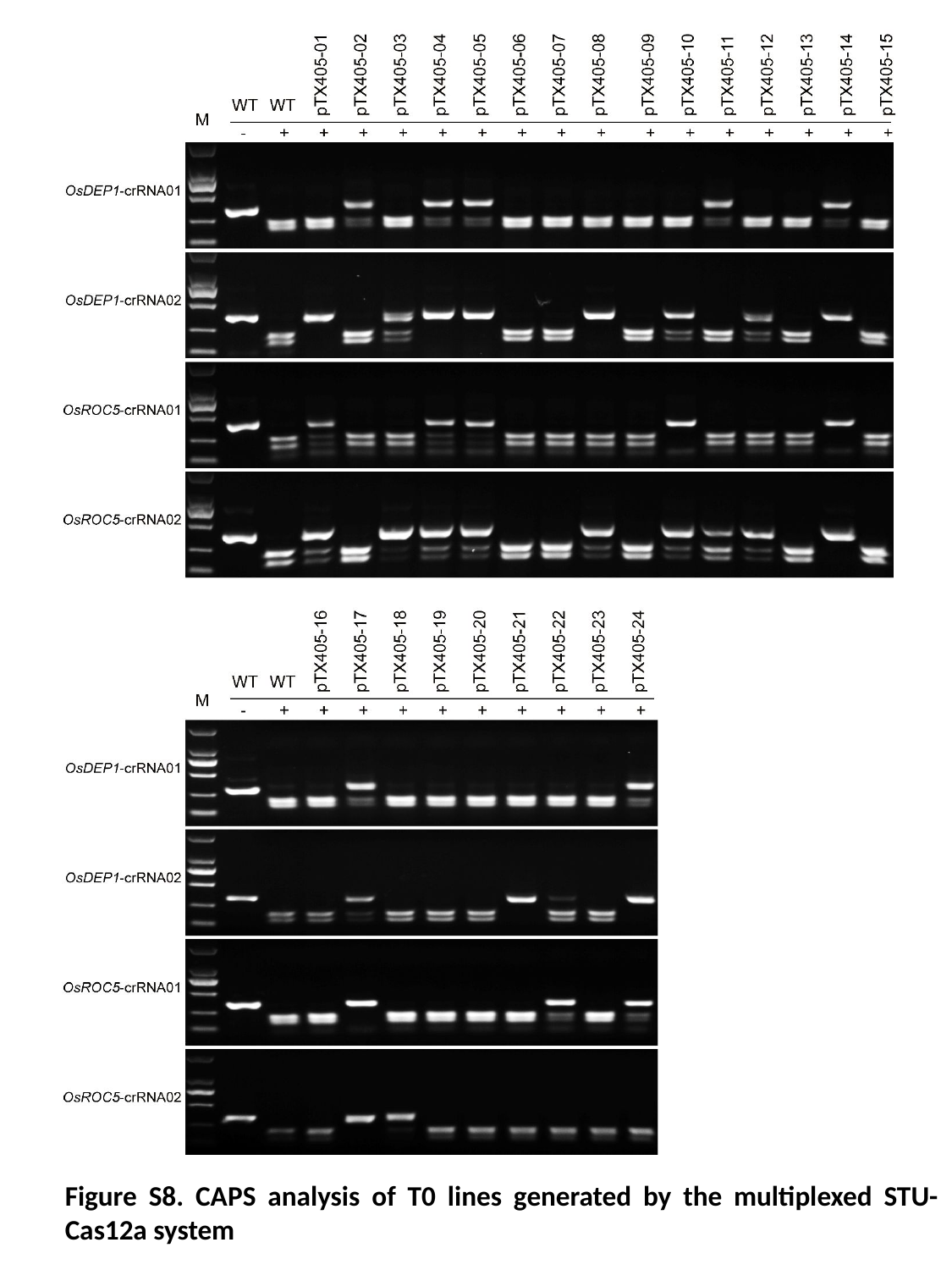

Figure S8. CAPS analysis of T0 lines generated by the multiplexed STU-Cas12a system
